# Learning Climate Perceptions as a Determinant of Employability: An Empirical Study Among European ICT Professionals

**DOI:** 10.3389/fpsyg.2018.02471

**Published:** 2018-12-20

**Authors:** Claudia M. Van der Heijde, Beatrice I. J. M. Van der Heijden, Dora Scholarios, Nikos Bozionelos, Aslaug Mikkelsen, Olga Epitropaki, Izabela Marzec, Piotr Jędrzejowicz, Jan C. Looise, the Indic@tor Study Group

**Affiliations:** ^1^University of Amsterdam, Amsterdam, Netherlands; ^2^Institute for Management Research, Radboud University, Nijmegen, Netherlands; ^3^Open University of the Netherlands, Heerlen, Netherlands; ^4^Kingston University, London, United Kingdom; ^5^University of Strathclyde, Glasgow, United Kingdom; ^6^EM Lyon Business School, Écully, France; ^7^University of Stavanger, Stavanger, Norway; ^8^Durham University, Durham, United Kingdom; ^9^University of Economics in Katowice, Katowice, Poland; ^10^Gdynia Maritime University, Gdańsk, Poland; ^11^University of Twente, Enschede, Netherlands

**Keywords:** learning climate, psychological climate, employability, older workers, career stages, life-span perspective, multi-source ratings, ICT professionals

## Abstract

This study investigated the role of age in the relationship between perceptions of learning climate and self- and supervisor-rated employability among European Information and Communication Technology (ICT) professionals. The psychological climate for learning was operationalized by three indicators, namely the perceptions that employees have of the learning value of their job, supervisor support for learning, and the organizational support for learning. As hypothesized, a Structural Equation Model demonstrated that the relationship between age and perceptions of learning climate was negative. The model also showed a strong positive relationship between learning climate and self-reported and supervisor-rated employability. Furthermore, learning climate perceptions appeared important for employability irrespective of life or career stage. An explorative bootstrapping-based test suggested that older workers with managerial responsibilities profit less from psychological learning climate for self-reported and supervisor-rated employability than older workers at non-managerial levels. These findings have important implications for human resource practices that aim to increase lifelong employability.

## Introduction

As a result of aging and dejuvenization of the working population, the competitiveness of developed countries in the next few decades is forecasted to depend increasingly on the contribution of older workers (Shultz and Adams, 2007; Van der Heijden et al., 2009b). Constant change in the work environment and in job content also means that, increasingly, both older and younger workers are required to continuously develop and maintain their work-related skills and employability (Semeijn et al., 2015; De Vos and Van der Heijden, 2017). This paper addresses the potential positive effects for worker employability when organizations actively promote a climate of learning across various employee career stages.

Stimulating workers' employability appears to be advantageous for both employee and organizational outcomes (Fugate et al., 2004; Van Dam, 2004; Van der Heijde and Van der Heijden, 2006; Rothwell and Arnold, 2007; Crook et al., 2011; Van der Heijden et al., 2018). Van der Heijde and Van der Heijden's (2006) competence-based approach to employability is an elaboration of the resource-based view of the firm (Nordhaug and Grønhaug, 1994; Wright et al., 1994). According to this view, competences are one category of possible resources that enable firms to achieve performance and (sustained) competitiveness. Highly employable workers (Van Dam, 2004) are necessary for organizations to meet fluctuating demands for numerical and functional flexibility (Valverde et al., 2000). When an organization encourages individual development and change its employees are better able to meet new or anticipated demands (see for instance: Argyris and Schön, 1978; Senge, 1990; Fugate et al., 2004; Rothwell and Arnold, 2007).

For the individual worker, employability is defined as the ability to obtain a job and to keep employed, within or outside one's current organization, for one's present or new customer(s), and with regard to future prospects (Van der Heijde and Van der Heijden, 2006) (see also Forrier and Sels, 2003; Fugate et al., 2004; Rothwell and Arnold, 2007). Increasingly, domain-specific occupational expertise is insufficient to guarantee positive work outcomes during the course of one's entire career. Rather, a broad competence package or a high level of *employability* that enables workers to cope with fast changing job requirements is needed. For an organization to be attractive to employees, it should provide lifelong and challenging learning opportunities: that is, chances to improve existing knowledge and skills and to develop new ones. Fortunately, several organizational policies and practices can be utilized to promote growth and learning behavior and to prevent obsolescence (e.g., Borghans et al., 2006; Bartram and Roe, 2008; Fouarge et al., 2009; Nikolova et al., 2016), herewith introducing the concept of learning climate.

Besides a possible direct effect on competence development, the way that employees perceive learning climate also potentially influences competence development indirectly via outcomes, such as job satisfaction, learning motivation (De Lange et al., 2005), and feelings of job security. Kooij's (2010) research also suggested that formal development practices are equally important for older and younger workers, in the light of the enhancement of positive employee outcomes, such as affective commitment, job satisfaction, and motivation to continue to work. For older workers, specifically, several studies (e.g., Boerlijst et al., 1993; Rhebergen and Wognum, 1997; Tikkanen et al., 2002; Borghans et al., 2006; Maurer, 2007) have already indicated that organizations generally expend less energy optimizing psychological learning climate. Notwithstanding the considerable amount of literature stressing the importance of learning climate, there is limited empirical research on the predictive validity of learning climate for employability development throughout the life span.

In this paper, we elaborate a theoretical framework focused on the age-learning climate–employability relationship and a life span perspective, and develop three hypotheses to test if learning climate perceptions really decrease with age. We then present a study of European Information and Technology (ICT) professionals designed to examine these hypotheses.

## Theoretical Background and Formulation of Hypotheses

### Psychological Learning Climate and Employability at Different Career Stages

Derived from the concept of *psychological* climate (indicating perception, e.g., Parker et al., 2003), and adapted from Nikolova et al. (2016, p. 259) we use the following definition of psychological learning climate: The *individual* perception of an employee of organizational policies and practices aimed at supporting employees' learning behaviors. In this empirical study, we will use a conceptualization of psychological climate that acknowledges situational constraints (Parker et al., 2003 after Jones and James, 1979), based on several work domains. This approach has the potential to provide organizations with practical starting points for stimulating learning climate experiences. In this section, we consider three themes associated with learning climate with respect to employee age, for which so called situational constraints for learning might occur: the learning value of the job, supervisor support for learning, and organizational support for learning (see also D'Amato and Zijlstra, 2008).

#### Learning Value of the Job

A job can be interpreted as a learning environment (e.g., Sims, 1983), and is the best preventive measure against obsolescence or plateauing (Rothman and Perrucci, 1970). The extent to which the job contributes to professional advancement is labeled the learning value of the job for the worker (Boerlijst et al., 1993, p. 57). This learning value depends on key features of the work, such as high quality job assignments and the degree of challenge and growth potential, especially with regard to the ability to utilize one's knowledge and skills. Supervisors fulfill a crucial role with regard to the assignment of challenging tasks and activities (Van Vianen et al., 2011). However, Maurer (2007) found that older workers more often receive routine tasks rather than complex and challenging job assignments that stimulate development (p. 168), herewith endangering their future employability (Van der Heijden et al., 2009a). Job content does not grow automatically with the expertise of older workers.

Boerlijst et al. (1993) found that the relatively low learning value conveyed by many jobs, especially among employees over 50, is a considerable problem. The percentage of employees in jobs offering too few opportunities for new learning experiences and, more specifically, for acquiring new expertise, was very high (reaching 45% and higher for jobs of medium and higher levels of education: ISCED level 3 and higher, see OECD). Furthermore, jobs with high work pressure, often do not leave room for new and challenging assignments. Thus, a contemporary challenge for organizations is to design jobs which facilitate continuous learning for all employees, while safeguarding efficiency and value for the employing organization or department (Van der Heijden, 2001, 2002). Furthermore, jobs should to a certain extent offer the space for workers to select their own tasks and challenging assignments (De Pater et al., 2009) in the context of their career development.

#### Supervisor Support for Learning

Supervisors can endanger the mobility and employability of their employees (Boerlijst et al., 1993; Boerlijst, 1994) by focusing on short-term goals to the exclusion of employees' long-term career goals. However, organizational policies and practices can enable supervisors to promote the development of occupational expertise. Concretely, supervisors can be empowered to stimulate employees to participate in training and development programmes, to exchange information, and to think about their future career steps. A basic requirement for growth is the formulation of a realistic and attainable individual career development plan by the employee, together with the supervisor and the HR department, where present (Stickland, 1996).

Maurer (2001), however, concluded that there was a systematic decrease of supervisory support for learning and development with employee age, for example, by withholding older workers from difficult job assignments, positions, and training experiences (for similar findings see also Rhebergen and Wognum, 1997). Maurer also showed that this decline in support negatively affects older workers' mastery experiences and their perceived self-efficacy for development. Van Vianen et al. (2011) demonstrated how such reluctance was perceived by employees. These authors found a negative relationship between employee age and willingness for further development among employees that perceived little developmental support from their supervisors, and whose supervisors perceived them to have a learning avoidance orientation.

Overall, studies examining the use of HRM practices for mature workers have found that few employers seem to encourage age awareness for their managers (Armstrong-Stassen, 2008). Such findings reinforce the importance of age awareness training which aims to promote managers' recognition of age bias, awareness of the needs of mature workers, and a change in attitude toward mature workers (Griffiths, 1997; Goldberg, 2005; Hedge et al., 2006).

#### Organizational Support for Learning

Organizational support for learning extends beyond the immediate job content of the worker, and his/her immediate supervisor. Unfavorable working conditions, situational constraints, and too little time to practice beyond the scope of “normal” work are problems confronting many employees (Peters and O'Connor, 1980; Paoli, 1997; Paoli and Merllié, 2001; Tikkanen, 2006). An increase in work intensification does not allow time or opportunities for experimentation or work outside one's immediate domain (Parent-Thirion et al., 2007). Concretely, important organizational support for learning comprises time (considering present high work pressures), support from the team wherein one is employed, and the experience of ample opportunities to develop (see Bartram et al., 1993).

*Time* enables workers to do their job properly and to learn effectively. Having time to think, practice, keep up with changes, and having time to talk things through with colleagues and line managers is important for employees' development. Time for self-regulation in learning is necessary to set learning goals, develop action plans for learning and seek feedback, and to evaluate learning results. Taris and Kompier (2004) argued that workers under time pressure tend to fall back on routine chores with no learning as a result. Availability of time might even be of higher importance for older workers' learning new knowledge and skills, due to the relative deterioration of fluid intellectual capabilities (Kanfer and Ackerman, 2004; Ng and Feldman, 2008, p. 400 and p. 403; cf. Baruch and Bozionelos, 2011).

A learning-provoking *team style* comprises a working environment with ample opportunities to learn from colleagues with expertise, who are supportive, caring and willing to help each other, and who are willing to share information and work. Under these circumstances, team members are seen as knowing their own limitations and as being willing to admit them. The importance of co-worker support for development has been underlined (Noe and Wilk, 1993; see for instance Maurer and Tarulli, 1994). Access to new information is critical, and much of the technical information needed by professionals comes from interactions with colleagues, for example by participation in social networks (e.g., Škerlavaj et al., 2010). For this reason, organizations should pay closer attention to information dissemination systems, and to the structure of these systems.

The fact that older workers are likely to perceive less support from their team-mates might be a result of their tenure and years of experience: older workers are more likely to be moved up in the hierarchy to be leaders or managers, and perceive less support from their boss or co-workers (Kawakami and Fujigaki, 1996). Another hindering factor for older workers concerns the difficulty of adapting to workplace changes, such as the requirement to adequately function within the setting of work teams (Yeatts et al., 2000), while being used to working individually. Moreover, older workers may experience problems in their collaboration with younger counterparts.

Employees reporting more *opportunities to develop*, perceive their workplace as providing opportunities to learn new skills and to do a variety of work. They see scope for creativity and opportunities for being a novice outside their own work domain. They have an awareness of what learning materials and options exist, and are involved in the discussion of plans and policies for change (both with regard to their work as well as the organization, in a broader sense). However, previous research has indicated that older workers in technology-intensive environments rarely described that they encountered totally new situations (Tikkanen, 2002). Based on the foregoing, we formulated our first hypothesis on the relation between employee age and all three discussed dimensions of learning climate.

H1: There exists a negative relationship between employee age and employees' perceptions of learning climate.
H2: There exists a positive relationship between employees' perceptions of learning climate and self-reported and supervisor-rated employability.

### Different Operationalizations of Age: Different Life and Career Stages

According to Kanfer and Ackerman (2004), the assumption of a general decline with age is simplistic, misleading and based on stereotyping (Pazy, 1996; Baruch and Bozionelos, 2011; see also Arnold and Clark, 2016). The authors presented a life-span perspective with regard to adult development and work motivation, including changes with regard to cognitive abilities, personality, affect, interests, and values with aging. “Work motivation in midlife and later years follows the same basic principles as work motivation in young adulthood—namely, the allocation of personal resources to work behaviors that build on competencies, promote a sense of self-efficacy and self-concept, and offer opportunities for the attainment of desired outcomes” (p. 455). Older workers can contribute in a different way and continue to be motivated for work and development. On top of that, there is more variability in work performance within age groups than between age groups (e.g., Ilmarinen, 2001 with regard to physical work capacity).

The meta-analysis of Ng and Feldman (2008) demonstrated that age was largely unrelated to core task performance, creativity, and performance in training programs. The weak negative relationship between age and motivation for training and career development activities found by Ng and Feldman (2012) in their follow-up meta-analysis, might be attributable to: (a) greater resistance and fear of failure because of deterioration of fluid intellectual capabilities; (b) lower perceived incentive with regard to return of investment; or (c) a self-fulfilling prophecy with regard to being offered less developmental activities. Since motivation to learn on the job does not appear to decrease with age (De Lange et al., 2005), learning climate is interpreted as an important predictor, both for older and younger workers. Although different age groups are expected to flourish with different learning climate profiles and are likely to demonstrate different patterns of competences throughout the life-span, age is approached as a continuous variable, since employees' development is variegated and does not occur at the same pace.

To examine the influence of age on the learning climate-employability relationship it is important to include different operationalizations of age, since calendar age serves only as a proxy for age-related processes (Kanfer and Ackerman, 2004; De Lange et al., 2010). Individuals may be at a different life- and career-stage, despite being the same calendar age. Approaches to the operationalization of age include: (a) performance-based or functional approaches (e.g., work ability or health measures); (b) psychosocial or subjective approaches (e.g., age norms applied to an individual with respect to an occupation, company, or society; cf. Kaliterna et al. (2002); (c) organizational approaches (e.g., job tenure, career stage); and (d) life-span approaches (e.g., family status or life stage measures) (De Lange et al., 2010, p. 943). This is also in line with Super's (1990) concept of recycling through the stages of adult career development, implying that career and life phases do not have a strict relation with chronological age and are flexible.

Working life nowadays consists of repeated cycles of learning (Hall and Mirvis, 1995; Baruch and Bozionelos, 2011). By including different career and life stage characteristics, herewith our model represents a broader conceptualization of the concept of age. As such, our approach is expected to lead to a better understanding of the age-learning climate-employability relationship. Specifically, we hypothesize that learning climate perceptions will be important for employability irrespective of life or career stage. This implies that including different career and life stage characteristics into the model, besides age, will not alter the previously hypothesized relationship between learning climate and employability.

H3: Learning climate perceptions will be important for employability irrespective of life- or career-stage.

## Methods

### Participants and Procedure

This study was carried out among pairs of ICT professionals and their immediate supervisors working in small- and medium-sized enterprises (SMEs) in seven countries (Germany, Greece, Italy, the Netherlands, Norway, Poland, and the United Kingdom). ICT professionals were defined as employees involved in the conception, development, implementation, and maintenance of software products and services (Van der Heijden et al., 2005). ICT professionals were estimated to be an eligible research population with regard to learning climate, since developments are very fast in the ICT industry (Scholarios et al., 2008; Marzec et al., 2009). The selection of employees excluded persons with low levels of education (not having attained ISCED level 3, see OECD), to be able to produce useful and comperative data with respect to future potential organizational change. This was a prerequisite to enable comparison of current and future workers, especially with regard to older workers (after ~20 years) in light of growing complexity and increasing level of difficulty of future jobs and concomitant rising required educational levels (see also Van der Heijde and Van der Heijden, 2006, p. 457).

We used two data collection methods: (1) an online questionnaire with immediate personalized feedback; and (2) a paper-and-pencil questionnaire, to increase the response rate in case a permanent internet connection in companies was less common. Informed consent was obtained by virtue of survey completion. Nevertheless, all employees received extensive information about the study before deciding to participate and received personalized confidential feedback after completion. They could also withdraw from the study at any time. The immediate supervisors were asked to respond to a shorter questionnaire, and were instructed to indicate how employable their subordinates were on a respective scale. The advantage of the use of multi-rater (or multi-source) performance ratings is that different evaluation perspectives add incremental validity to the assessment of individual performance (see for instance Borman, 1997). In line with the work by (Facteau and Craig (2001), p. 215) on performance appraisals, we expected an equivalent factor structure of the employability construct among the rater groups (employees and supervisors) (see also Van der Heijde and Van der Heijden, 2006; Van der Heijden et al., 2009b).

It was accepted that achieving a sufficient sample size would depend on elements of convenience sampling, utilizing personal contacts and networks. An examination of the final respondent characteristics indicated that we did, indeed, achieve a representative sample of ICT professionals in most countries, comparing the obtained data in each country with the three sampling criteria in the defined sampling frame: (1) the geographical regions in each country where most ICT activity occurs; (2) the ICT industry subsector in which ICT professionals are employed, including both ICT suppliers and ICT users; and (3) the size of the organizations in which they were employed, taking into account that the majority of the population of ICT organizations comprises SMEs (Van der Heijden et al., 2005).

Our final sample consisted of 967 pairs (response rate was 69% for employees, and 63% for supervisors across countries for the web-based survey and around 25% for the paper-and-pencil versions). The employees' sample included 694 men (71.8%) and 273 women (28.2%). Mean age was 34.50 years (*sd* = 8.29), with an average of 9.82 years (*sd* = 8.07) on the labor market, of which 7.21 (*sd* = 5.80) was as an ICT professional, and average length of service for the organization was 4.72 years (*sd* = 4.45). In total, 728 of the supervisors were men (75.3%) and 239 were women (24.7%). Their mean age was 41.53 years (*sd* = 7.82).

### Measures

The questionnaire was used in a large European study during which several precautions were undertaken to increase the measurement equivalence of the factors included across the participating countries (e.g., Van de Vijver and Leung, 1980): (1) extensive testing concerning the item formulation of all instruments among five employees, five supervisors, and two communication specialists in the Netherlands; (2) instructions regarding cross-checking of the self-ratings with the ratings of the supervisor (on employability) as well as guaranteeing anonymity (see also Mabe and West, 1982); (3) extensive pre-testing of the questionnaire in a pilot following the translation back-translation methodology, herewith solving possible language and linguistic problems, and aimed at enhancing user-friendliness; (4) a needs analysis amongst ICT employers was used as a cross-validation for the rationale of incorporating the study's variables, with societal and labor market needs.

Employability was assessed with Van der Heijde and Van der Heijden's (2006) domain-independent (or generic) instrument that contains five dimensions: (1) occupational expertise (15 items); (2) anticipation and optimization (8 items); (3) personal flexibility (8 items); (4) corporate sense (7 items); and (5) balance (9 items). Construct validity for the employability instrument, including the second-order structure of the concept, equivalent factor structure over the different rater groups (employees and supervisors), and predictive validity were demonstrated using structural equation modeling and multi-trait-multimethod analysis (Van der Heijde and Van der Heijden, 2006; Bozionelos et al., 2016). Examples were: “By virtue of my experience with him/her, I consider him/her … competent to be of practical assistance to colleagues with questions about the approach to work” (ranging from “not at all” to “extremely”) (*occupational expertise*), “(S)he is … focused on continuously developing him/herself” (ranging from “not at all” to “a considerable degree”) (*anticipation and optimization*), “(S)he adapts to developments within the organization …” (ranging from “very badly” to “very well”) (*personal flexibility*), “(S)he manages to exercise … influence within the organization” (ranging from “very little” to “a very great deal”) (*corporate sense*), and “The time (s)he spends on his/her work and career development on the one hand, and his/her personal development and relaxation on the other are…evenly balanced” (ranging from “not at all” to “a considerable degree”) (balance). The item sets for the employees and the supervisors are nominally identical and all scored on a six-point rating scale.

Learning climate was assessed on the individual (psychological) level using three scales: (1) learning value of the job; (2) supervisor support for learning; and (3) organizational support for learning. *Learning value of the job* was assessed with Van der Heijden's instrument (Van der Heijden et al., 2005; Van der Heijden and Bakker, 2011). A sample item was: “My job enables me to further develop my talents.” The item sets for the employees are all scored on a six-point rating scale (ranging from “strongly disagree” to “strongly agree”). *Supervisor support for learning* was assessed by means of a thoroughly validated, five-item instrument (Van der Heijden et al., 2005, 2010) An example item was: “My supervisor has taken into account my age and capacity when assigning me new tasks and responsibilities during the last year.” The item sets for the employees are all scored on a six-point rating scale (ranging from “strongly disagree” to “strongly agree”).

*Organizational support for learning* was measured with the multivariate learning climate questionnaire (LCQ) of Bartram et al. (1993) that provides measures of three aspects of learning opportunity: time (seven items, sample item “in some parts of the job there is not enough time to keep up with changes,” team style (nine items, sample item “if we ask for help it is given”) and opportunity to develop (six items, sample item “our ideas for changes are welcomed by management.” Response format was five-point (“never true” to “always true”).

Since in the literature on the subject there is no agreement how different indicators of career stage and life phase representing age should be operationalized (Lynn et al., 1996; De Lange et al., 2010; Kooij and Boon, 2018), we decided to apply multiple indicators (Super, 1990; Lam et al., 2012). General perceived health, representing a functional approach of age, was measured with a 5-item general health scale of the SF-36 Health Survey (Ware and Sherbourne, 1992). A sample item was “My health is excellent.” All items, except the first one, are scored on a five-point rating scale ranging from: (1) definitely false, to (5) definitely true. Work role [measured as managerial responsibilities (yes/no)] was included as an important indicator with regard to career stage of ICT professionals (see also Kappelman et al., 2016) thereby representing a subjective approach to age (age norms). Furthermore, we included ICT professional tenure (in years) (Lam et al., 2012), and length of supervisory relationship (in months), as operationalizations of an organizational approach of age. Family status (number of children) represented a life-span operationalization of age.

Highest educational qualification of the employee and age of the supervisor were used as control variables. Highest educational qualification was measured with one item and the following scale anchors: High school or equivalent; College (some university); Bachelor's degree (or recognized equivalent); Master's degree (or recognized equivalent); and Doctorate (PhD). According to Ostroff and Atwater (2003), gender of the supervisor affects compensation levels but not performance ratings. Therefore, we did not control for supervisor gender.

### Ethical Considerations

As regards ethical guidelines, full review and approval by the Ethical Committee was not required, according to national legislation, since there was no impairment of medical integrity and no interventions were performed (Wet Bescherming persoonsgegevens, 2000). Standards from the American Psychological Association and the British Psychological Society were followed as well as standards from the European Commission (within the Fifth Framework) and NWO: The Netherlands Organization for Scientific Research. Furthermore, the study was led by a psychologist (Beatrice I.J.M. van der Heijden), registered at the NIP: The Dutch Association of Psychologists, who initiated and coordinated the study.

## Results

### Descriptive Statistics

Cronbach's α coefficients of all employability dimensions were in the acceptable range; the self-ratings ranged from 0.78 to 0.93, and the supervisor ratings from 0.80 to 0.95, see Table 1. The supervisor-rated employability dimensions correlated highly (*r* ≥ 0.66), while the self-rated employability dimensions correlated somewhat lower (*r* ≥ 0.37). The consistency between supervisor-ratings and self-ratings for the same employability dimensions ranged from 0.21 to 0.53. All learning climate perception measures demonstrated good internal consistencies, with Cronbach alphas ranging from 0.77 to 0.85.

**Table 1:** Means, standard deviations, reliability coefficients (Cronbach's α on the diagonal), and correlations between the model variables, *N* = 967.

	**Mean**	**SD**	**1**	**2**	**3**	**4**	**5**	**6**	**7**	**8**	**9**	**10**	**11**	**12**	**13**	**14**	**15**	**16**	**17**	**18**	**19**	**20**	**21**
(1) Age employee	34.50	8.29	–																				
(2) Highest Educ. Qual. employee	2.56	1.13	−0.06	–																			
(3) ICT Professional tenure (years)	7.21	5.80	0.70	−0.11	–																		
(4) Health	3.77	0.72	−0.22	0.13	−0.09	(0.79)																	
(5) Age supervisor	41.53	7.82	0.29	0.01	0.16	−0.05	–																
(6) Length supervision (months)	34.95	26.63	0.30	−0.01	0.26	−0.11	0.33	–															
**LEARNING CLIMATE PERCEPTIONS**																							
(7) Learning value	4.20	0.84	−0.11	0.09	−0.16	0.24	0.01	0.07	(0.85)														
(8) Supervisor support for learning	3.23	1.08	0.03	−0.03	0.06	0.05	−0.02	−0.05	0.12	(0.80)													
(9) Time	3.06	0.63	−0.13	0.07	−0.15	0.19	0.01	−0.03	0.07	−0.01	(0.82)												
(10) Team	3.52	0.60	−0.14	0.06	−0.15	0.23	−0.02	−0.08	0.35	0.22	0.32	(0.84)											
(11) Opportunities	3.26	0.63	−0.07	0.04	−0.08	0.13	0.02	0.04	0.45	0.28	0.23	0.57	(0.77)										
**EMPLOYABILITY SUPERVISOR**																							
(12) Occupational expertise	4.58	0.76	−0.05	0.24	−0.06	0.24	−0.07	0.05	0.31	−0.01	0.18	0.20	0.26	(0.95)									
(13) Anticipation and optimization	4.19	0.82	−0.09	0.20	−0.11	0.21	−0.04	0.05	0.38	0.06	0.08	0.18	0.25	0.72	(0.89)								
(14) Personal flexibility	4.32	0.75	−0.07	0.21	−0.13	0.30	−0.03	0.03	0.37	−0.04	0.21	0.29	0.33	0.79	0.74	(0.87)							
(15) Corporate sense	4.19	0.90	0.05	0.18	0.03	0.19	0.04	0.14	0.32	0.07	0.06	0.19	0.30	0.74	0.74	0.72	(0.88)						
(16) Balance	4.28	0.72	−0.12	0.17	−0.12	0.26	−0.07	0.06	0.32	0.03	0.13	0.22	0.28	0.69	0.66	0.67	0.67	(0.80)					
**EMPLOYABILITY EMPLOYEE**																							
(17) Occupational expertise	4.62	0.62	0.02	0.25	0.10	0.35	−0.07	0.00	0.26	0.11	0.17	0.31	0.29	0.53	0.36	0.40	0.34	0.39	(0.93)				
(18) Anticipation and optimization	4.05	0.72	−0.12	0.16	−0.07	0.19	−0.03	0.02	0.41	0.26	0.04	0.28	0.33	0.23	0.40	0.27	0.21	0.23	0.45	(0.82)			
(19) Personal flexibility	4.27	0.61	−0.03	0.10	−0.02	0.37	0.00	−0.02	0.39	0.17	0.18	0.41	0.42	0.36	0.32	0.44	0.28	0.32	0.63	0.54	(0.79)		
(20) Corporate sense	4.00	0.78	0.13	0.13	0.17	0.18	0.06	0.11	0.33	0.28	−0.02	0.35	0.48	0.26	0.24	0.28	0.37	0.24	0.50	0.55	0.55	(0.81)	
(21) Balance	4.08	0.65	−0.06	0.12	−0.05	0.27	0.03	0.06	0.34	0.18	0.27	0.41	0.43	0.28	0.23	0.28	0.25	0.38	0.46	0.42	0.48	0.44	(0.83)

*Correlations between 0.06 ≤ r ≤ 0.08 are significant at p < 0.05 while correlations r ≥ 0.09 are significant at p < 0.01*.

### Preliminary Analyses

Besides chronological age, we explored different operationalizations of age in the model to do full justice to the earlier formulated life-span perspective (Kanfer and Ackerman, 2004; De Lange et al., 2010): perceived health, organizational tenure, ICT professional tenure, length of supervision, work role (i.e., managerial responsibilities), and family status (number of children). Regression analyses of these variables on each of the employability dimensions separately (both supervisor and employee version), showed that perceived health, ICT professional tenure, length of supervision (in months), and work role (i.e., managerial responsibilities) were significantly related to both employability supervisor ratings and self-ratings (see Table 2). Only factors that appeared to be significantly related to the outcome measures were subsequently included in further analyses.

**Table 2:** Results of the hierarchical regression analyses with socio-demographic characteristics.

	**Supervisor**					**Employee**				
**Step**	**Occupational**	**Anticipation and**	**Personal**	**Corporate**	**Balance**	**Occupational**	**Anticipation**	**Personal**	**Corporate**	**Balance**
	**expertise**	**optimization**	**flexibility**	**sense**		**expertise**	**optimization**	**flexibility**	**sense**	
Country	0.06	−0.03	−0.01	0.01	0.02	0.05	−0.05	−0.07a	0.03	0.04
Gender employee	−0.03	−0.04	−0.04	0.00	−0.04	−0.02	0.04	0.01	0.09b	0.05
Highest educational qualification	0.18c	0.16c	0.15c	0.14c	0.12c	0.21c	0.15c	0.06a	0.10b	0.09b
Organizational tenure	0.05	−0.03	0.00	0.02	0.07	0.04	−0.12b	−0.07	−0.06	0.02
ICT professional tenure	−0.12a	−0.12a	−0.20c	−0.08	−0.16b	0.08	0.05	−0.03	0.10a	−0.03
Work role (i.e., managerial)	−0.10b	−0.09b	−0.10b	−0.12c	−0.05	−0.10b	−0.08a	−0.10b	−0.25c	−0.01
Health	0.22c	0.18c	0.29c	0.19c	0.25c	0.32c	0.14c	0.37c	0.18c	0.25c
Family status (Children)	−0.03	0.01	−0.01	0.02	−0.01	−0.04	−0.02	−0.01	0.01	−0.01
Gender supervisor	0.08a	0.09b	0.14c	0.07a	0.06a	−0.03	0.03	0.05	−0.02	0.09b
Length of supervision	0.12b	0.15c	0.10b	0.16c	0.15c	0.01	0.10b	0.03	0.08a	0.07a
Age employee	0.07	0.01	0.12a	0.09	−0.01	0.05	−0.08	0.10a	0.08	−0.01
Age supervisor	−0.11b	−0.07a	−0.06	−0.04	−0.09b	−0.08a	−0.02	0.01	−0.01	0.03

*Gender: female = 0, male = 1, a p < 0.05; b p < 0.01; c p < 0.001*.

### Test of the Age–Learning Climate Perceptions–Employability Relationship

**Figure 1:**
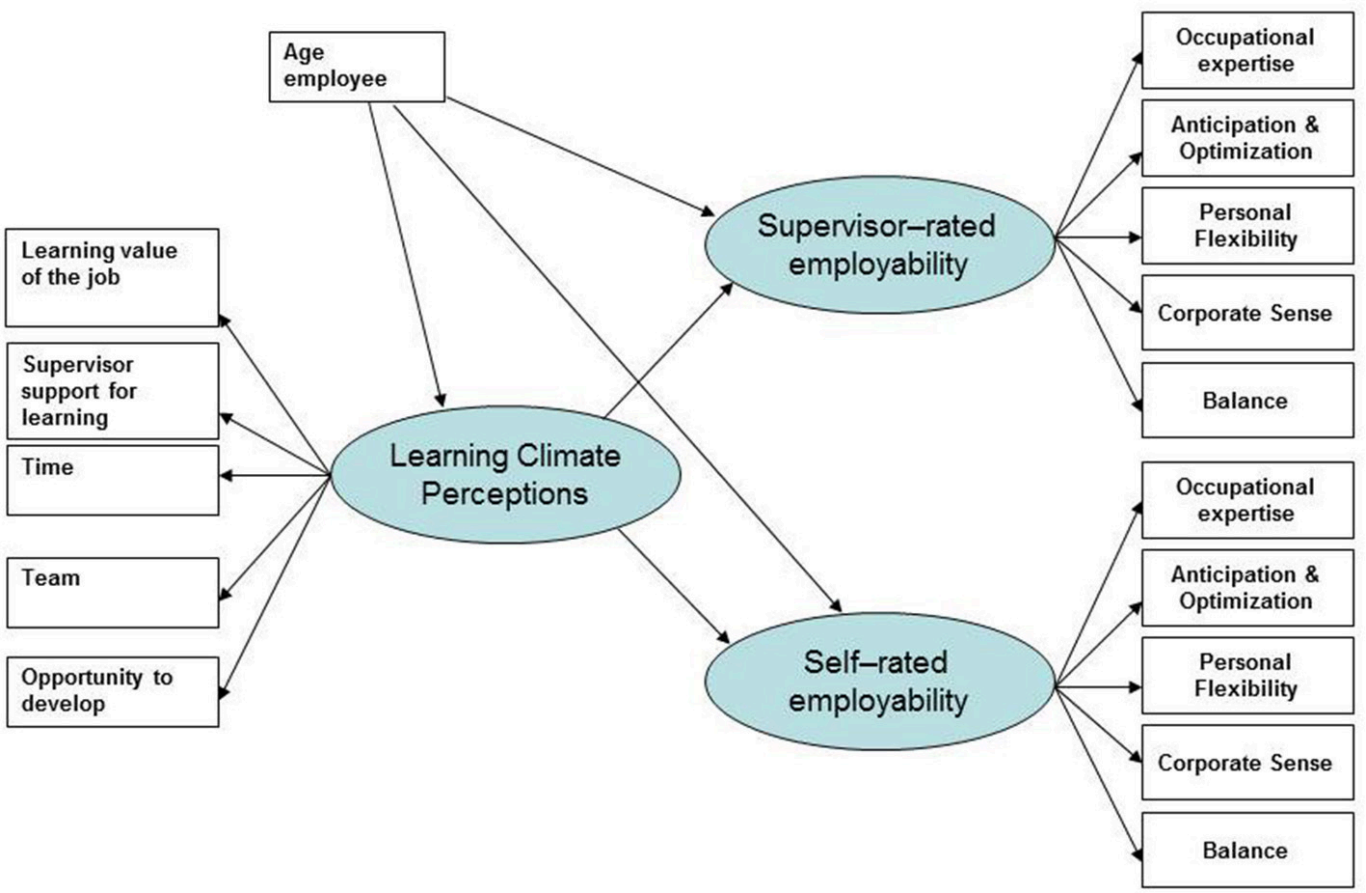
A model of employability, enhanced by learning climate perceptions.

We tested our first and second hypotheses by means of Structural Equation Modeling (SEM) using the maximum likelihood method with the AMOS computer program (Arbuckle, 2003). Both learning climate perceptions and self-reported and supervisor-rated employability were included as latent endogenous factors (see Figure 1). The SEM analysis was conducted with the mean scores of the scales, instead of the scale items. Previous results of Confirmatory Factor Analysis (Van der Heijde and Van der Heijden, 2006; Van der Heijden et al., 2009b; Bozionelos et al., 2016) supported the suggested factor structure of employability. The measurement errors of the parallel dimensions (supervisor and employee version) were allowed to correlate.

To test the fit between the proposed model and the data, the traditional χ^2^-value, the Comparative Fit Index (CFI), the Normed Fit Index (NFI) and the Root Mean Square Error of Approximation (RMSEA) were calculated. A CFI ≥ 0.90, NFI ≥ 0.90, and a RMSEA ≤ 0.08 indicate a reasonable fit between the model and the data. The model for the total sample appeared to have a reasonable fit (χ^2^ = 723.89, df = 116, CFI = 0.93, NFI = 0.91, RMSEA = 0.07, see Model 1, Table 3).

**Table 3:** Goodness of fit indices for proposed models.

**Model**	** χ^2^**	***df***	**CFI**	**NFI**	**RMSEA**
(1)	723.89	116	0.93	0.91	0.07
Null	8, 450.63	171	0.00	0.00	0.22
(2)	1, 408.88	351	0.89	0.86	0.06
Null	10, 029.17	462	0.00	0.00	0.15
(3)	1, 272.45	336	0.90	0.88	0.05
Null	10, 144.20	420	0.00	0.00	0.15

The significant structural path showed that age was negatively related to learning climate perceptions (β = −0.13, *p* < 0.001), providing support for Hypothesis 1. Furthermore, learning climate perceptions were positively related to both supervisor-rated employability and self-reported employability (β = 0.43, *p* < 0.001 and β = 0.75, *p* < 0.001), providing support for Hypothesis 2 (for the supervisor ratings not as strongly positive as for the self-ratings). The proportion of explained variance in this model was 23% for supervisor-rated employability and 58% for self-rated employability.

### Test of the Age–Learning Climate Perceptions-Employability Relationship, Including Different Career-and Life-Stage Characteristics Into the Model

**Figure 2:**
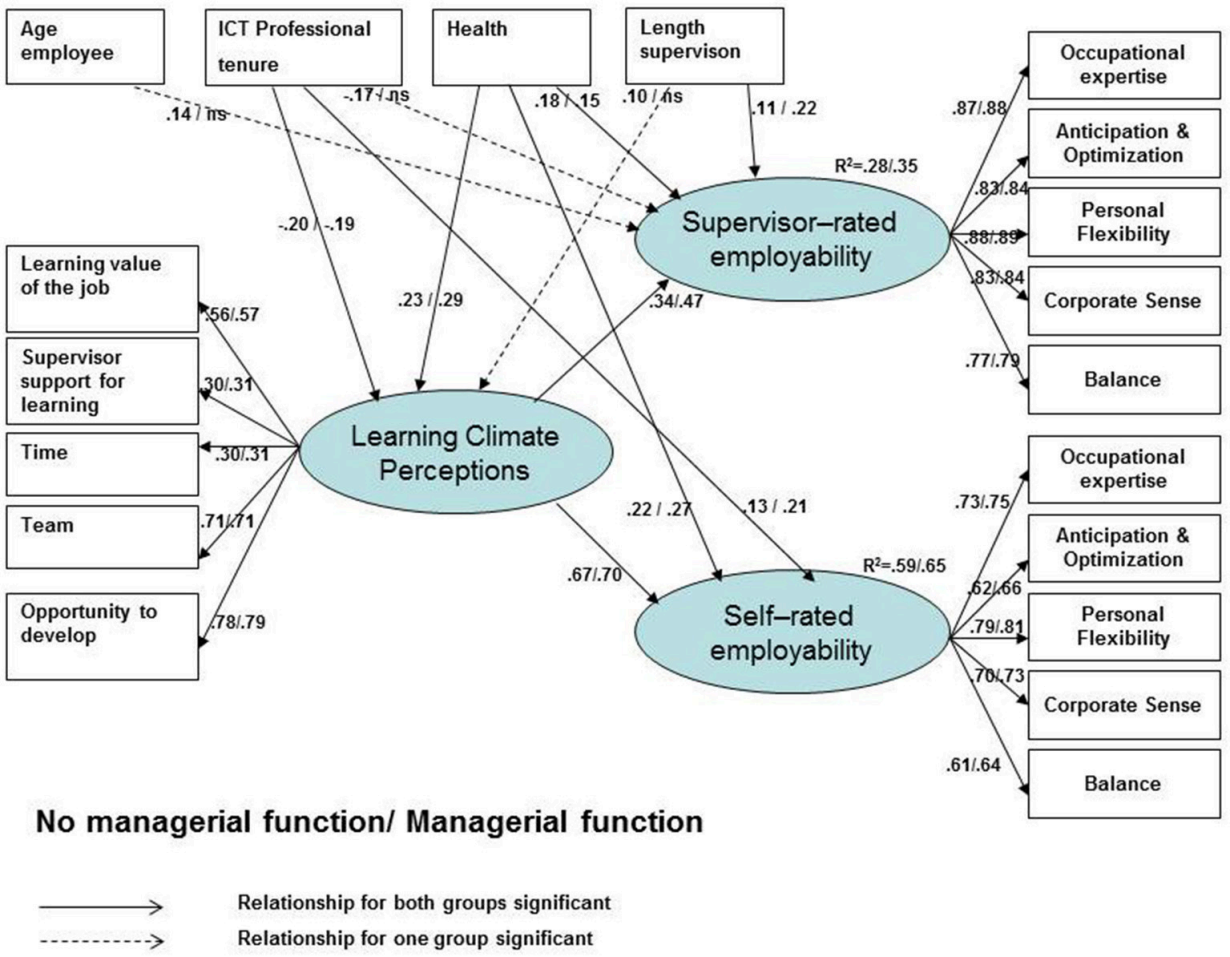
A model of employability, enhanced by learning climate perceptions related to different career and life stage characteristics.

When testing Hypothesis 3, we performed a SEM analysis testing our model of the age—learning climate perceptions—employability relationship, adding perceived health, ICT professional tenure, length of supervision (in months), and work role (i.e., managerial responsibilities) into the model (see Model 2, Table 3, and Figure 2). The model had a satisfactory fit to the data, χ^2^ = 1408.88, df = 351, CFI = 0.89, NFI = 0.86, RMSEA = 0.06, given the large number of items we used (Patterson et al., 2005).

More specifically, for the category without managerial responsibilities we found that age was no longer significantly related to learning climate perceptions and employability (self-ratings). Age was related positively and significantly to supervisor-ratings of employability (β = 0.14, *p* < 0.01). The learning climate—employability relationships remained unaltered (H3). Learning climate perceptions were significantly and positively related to both supervisor-ratings and self-ratings of employability (β = 0.34, *p* < 0.001 and β = 0.67, *p* < 0.001 respectively).

For the category with managerial responsibilities we found comparable results: age was no longer significantly related to learning climate perceptions or employability (both supervisor and self-ratings). The learning climate—employability relationships remained unaltered (H3). Learning climate perceptions were significantly and positively related to both supervisor-ratings and self-ratings of employability (β = 0.47, *p* < 0.001 and β = 0.70, *p* < 0.001, respectively). These findings imply support for Hypothesis 3 that learning climate perceptions will be important for employability irrespective of life or career stage.

The proportion of explained variance in this model was 28% for supervisor-rated employability and 59% for self-rated employability for the category without managerial responsibilities, while it was 0.35 for supervisor-rated employability and 0.65 for self-rated employability for the category with managerial responsibilities. On an explorative basis we found significant indirect effects (with a 90% confidence interval) of age and all age/career-and life-stage variables via learning climate on supervisor and self-rated employability, using the bootstrapping-based test (MacKinnon et al., 2004) with 2000 bootstrap resamples (see Model 3 and Tables 3, 4).

**Table 4:** Exploration of indirect effects of age/ different career and life stage characteristics and learning climate perceptions on employability: standardized indirect effects and the associated 90% confidence intervals.

**Variable**	**Indirect effect**	**90% confidence interval**
**NO MANAGERIAL RESPONSIBILITIES**		
(1) Age -> learning climate perceptions -> employability supervisor ratings	0.004^*^	[−0.028, 0.039]
(2) Age -> learning climate perceptions -> employability self-ratings	0.008^*^	[−0.056, 0.076]
(3) ICT professional tenure -> learning climate perceptions -> employability supervisor ratings	−0.068^*^	[−0.108, -0.037]
(4) ICT professional tenure -> learning climate perceptions -> employability self-ratings	−0.135^*^	[−0.201, -0.069]
(5) Length supervision -> learning climate perceptions -> employability supervisor ratings	0.032^*^	[0.004, 0.064]
(6) Length supervision -> learning climate perceptions -> employability self-ratings	0.063^*^	[0.007, 0.120]
(7) Health -> learning climate perceptions -> employability supervisor ratings	0.078^*^	[0.051, 0.118]
(8) Health -> learning climate perceptions -> employability self-ratings	0.155^*^	[0.102, 0.215]
**MANAGERIAL RESPONSIBILITIES**		
(1) Age -> learning climate perceptions -> employability supervisor ratings	−0.011^*^	[−0.094, 0.071]
(2) Age -> learning climate perceptions -> employability self-ratings	−0.016^*^	[−0.133, 0.108]
(3) ICT professional tenure -> learning climate perceptions -> employability supervisor ratings	−0.087^*^	[−0.179, -0.004]
(4) ICT professional tenure -> learning climate perceptions -> employability self-ratings	−0.129^*^	[−0.272, -0.001]
(5) Length supervision -> learning climate perceptions -> employability supervisor ratings	−0.002^*^	[−0.053, 0.042]
(6) Length supervision -> learning climate perceptions -> employability self-ratings	−0.003^*^	[−0.078, 0.061]
(7) Health -> learning climate perceptions -> employability supervisor ratings	0.132^*^	[0.070, 0.218]
(8) Health -> learning climate perceptions -> employability self-ratings	0.196^*^	[0.104, 0.299]

* *p < 0.10*.

## Discussion

The results in this study justify taking a life-span perspective (Kanfer and Ackerman, 2004) on learning in organizations and stress the importance of approaching each worker as an individual in that respect. Learning climate ratings appeared to decline with age unless different career- and life-stage characteristics were taken into account. Moreover, adding these factors into our model led to a larger proportion of explained variance in the supervisor ratings and self-ratings of employability, especially for workers with managerial responsibilities. Furthermore, we found a stable positive relationship between learning climate perceptions and employability (supervisor ratings and self-ratings of employees).

Older employees without managerial responsibilities seem to profit from higher supervisor employability ratings. Another finding was that employees without managerial responsibilities with longer ICT professional tenure were given less positive employability ratings by their supervisors. A potential explanation for this finding is higher expectations of supervisors caused by a higher level of workers' experience. These effects were less pronounced for employees with managerial responsibilities (herewith indicating a so-called trend). In all instances, health was positively related to a better perceived learning climate and employability experience. Workers without managerial responsibilities with shorter ICT professional tenure and/or working longer with the same supervisor seem to experience better learning climates. That was not the case for workers with managerial responsibilities.

When exploring for indirect effects with the bootstrapping method, we found both age and the other career and life stage characteristics to be significant moderators of the psychological learning climate—employability relationship (Table 4). When trying to interpret these results, it seems as though older workers with managerial responsibilities profit less from the learning climate in the light of their employability contrary to older workers without managerial responsibilities. Adding to that, workers with longer ICT professional tenure also profit less from the learning climate for their employability (both with and without managerial responsibilities). Workers without managerial responsibilities who were supervised longer by their manager profit more from the learning climate for their employability contrary to workers with managerial responsibilities. All workers with higher perceived health profit more from the learning climate for their employability.

These findings underscore the importance of incorporating life phase factors when deciding about optimization of learning experiences within the organization. One possible explanation for the fact that workers with longer ICT professional tenure profit less from the learning climate for their employability could also be their lowered motivation for learning in that job environment, necessitating some form of internal or external stimulation (i.e., switch of tasks, roles, organization and/or sector).

Furthermore, additional research is necessary to understand the differences in learning climate experience for workers with or without managerial responsibilities. Workers with managerial responsibilities appeared to be a distinct group of workers that, at first view, seems to be less strongly influenced by life stage factors regarding their perceived learning climate and employability ratings. At second glance though, they seem to profit less from the perceived learning climate for their employability with increasing age (including the different life stage factors), implying that they may have specific needs for development. On a functional level, work content of workers with managerial responsibilities can differ substantially from workers without managerial responsibilities and they might need different stimuli to develop their careers. For instance, in the case of developing their transactional leadership styles into more transformational styles, factors such as personality and maturity come into play. Furthermore, older managers who have reached career plateaus can have different career orientations, being motivated more by subjective career success as opposed to younger managers that strive for more objective career success, such as upward career moves and salary increases (Bown-Wilson and Parry, 2013), The reason that older managers are less able to benefit from the psychological learning climate of the company for increasing their employability could be a reflection of their underlying work motivations.

We do not advocate a position-based approach that is prone to positive and negative discrimination; for instance, fostering a more positive learning climate for employees with a higher ranking in the organization, or for younger employees. We rather advocate a resource-based approach that is focused on equally developing competences of both younger and older workers (see also Tikkanen et al., 2002). Likewise, Maurer and Rafuse (2001), in their study on preventing age discrimination when managing the organization's development process, stressed the need for policies that imply allocation of developmental opportunities on an age-neutral, and individual-by-individual basis. This follows knowledge about large individual variability with regard to developmental potential, performance and career orientation among (older) workers (Ilmarinen, 2001; Kanfer and Ackerman, 2004; Bown-Wilson and Parry, 2013).

Organizations should invest energy into career development for all age and life phase groups, for instance, (older) managers and workers starting their retirement process. Such processes could include both formal and informal learning climate, such as training, job experiences, job rotation, supervisor support, supervisor support for learning, mentoring programs, and career and role change, including career coaching (e.g., Bown-Wilson and Parry, 2013). Parallel to that, organizations should remove constraints (e.g., time constraints) that have potential detrimental effects on the employee's motivation to learn and on learning self-efficacy. These would also include flexible working and flexible retirement policies for workers that want to slow down (Bown-Wilson and Parry, 2013). LePine et al. (2004) demonstrated relationships of challenge and hindrance stress on learning performance via motivation to learn (see also Mathieu and Martineau, 1997).

Improving the learning climate is also important in order to reduce job stress and to promote occupational health (Mikkelsen et al., 1998). A mismatch between individual learning climate perceptions and the averaged evaluation of learning climate in the rest of the working group was found to be an important source of stress. Mikkelsen et al. (1999) found that decision authority and learning opportunities appeared to have a specific and independent impact on subjective health, psychological functioning, coping style and organizational outcome variables. Finally, excluding older workers from learning climate optimization policies makes them vulnerable for skills obsolescence and job termination: it appears that there is a relationship between unemployment and mental health for this age group, and not for younger workers (Breslin and Mustard, 2003).

### Limitations and Recommendations for Further Research

First, our survey research design allows for response set consistencies to occur. Secondly, in order to increase certainty that the relationships we found occur in the sequence as hypothesized, longitudinal research is needed. Thirdly, research into the generalizability of our findings to other occupational settings is recommended. Not including country as a variable in the model was justified by our preliminary analyses. This result is also plausible considering that the first dominating culture of this sample is the ICT culture. Fourthly, including measures of learning self-efficacy and learning motivation (see e.g., Maurer et al., 2003) might contribute to our understanding of the relationships between age, learning climate perceptions and employability.

### Practical Implications

Enhancing learning climate experience in the firm (and department) is an essential component of investing in human competence through suitable HR policies and practices (Boxall, 1999; Van der Heijde and Van der Heijden, 2006). Secondly, improving the learning climate experience in organizations is important to keep aging workers in the labor force, thereby utilizing their carefully built-up expertise, as long as possible. Older workers who are less motivated and report low self-efficacy have already left the labor market instead of adapting to workplace changes (see for instance, Yeatts et al., 2000).

Companies can collect information on different aspects of the learning climate from their workers, both young and old, and on a group by group basis (both at organization and department level) thereby taking into account different life-span factors. Follow-up is possible on several levels. First, at management level, outcomes for different types of workers (e.g., younger/older, those with longer/shorter sectoral experience, those with managerial/no managerial responsibilities, those who are healthy/unhealthier) can be discussed within the management team, offering starting points for improvement of the learning climate experience. Secondly, at an individual level, workers can be encouraged to undertake action(s) to enhance their learning climate experience and to remove hindrances to learning. In order to be able to do this, they need some form of personalized confidential feedback, including some means they can use for action; for instance, writing a personal development plan and discussing it with their superior, putting their personal hiatus on the agenda of the weekly meeting, or discussing their personal learning climate issue with a colleague. The valid and reliable measurement instrument developed for this study is suitable for providing a thorough insight of a company's learning climate, and that of different sections, as well as for use as an HR tool to develop the individual worker.

## Author Contributions

All authors of this study were involved in all stages of the research process: conception and design, data collection and processing, analysis and interpretation of the data, and writing substantial sections of the paper.

### Conflict of Interest Statement

The authors declare that the research was conducted in the absence of any commercial or financial relationships that could be construed as a potential conflict of interest.
